# Immunoregulation of Quercetin and Kynurenic Acid on Human Umbilical Cord Mesenchymal Stem Cells Through Gene Expression of Aryl Hydrocarbon Receptor and Interleukin-6 in Hyperglycemic Milieu

**DOI:** 10.1155/sci/6612312

**Published:** 2025-06-28

**Authors:** Thi Sam Nguyen, Thi Thuy Ngan Nguyen, Thi Phuong Anh Nguyen, Tran Bao Chau Ha, Manh Cuong Nguyen, Syed Shadab Raza, Vinh Truong Do, Hoang Ha Chu

**Affiliations:** ^1^Graduate University of Science and Technology, Vietnam Academy of Science and Technology, 18 Hoang Quoc Viet, Cau Giay, Hanoi, Vietnam; ^2^Stem Cell and Gene Therapy Applied Research Center, Institute of Biotechnology, Vietnam Academy of Science and Technology, 18 Hoang Quoc Viet, Cau Giay, Hanoi, Vietnam; ^3^Institute of Natural Products Chemistry, Vietnam Academy of Science and Technology, 18 Hoang Quoc Viet, Cau Giay, Hanoi, Vietnam; ^4^Laboratory for Stem Cell and Restorative Neurology, Department of Biotechnology, Era's Lucknow Medical College Hospital, Era University 226003, Sarfarazganj, Lucknow, India

**Keywords:** aryl hydrocarbon receptor, human umbilical cord mesenchymal stem cell, kynurenic acid, quercetin, UC-MSC

## Abstract

Mesenchymal stem cells (MSCs) exhibit great promise for treatment applications because of their immunosuppressive properties. The aryl hydrocarbon receptor (AHR), which is a transcription factor that is activated via ligand, has a pivotal role in regulating the immune system and is involved in a range of immune-related disorders. However, hyperglycemia, the defining biochemical hallmark of diabetes, creates a chronically pro-inflammatory microenvironment that impairs the immunoregulatory effects of MSCs. In this study, we explored the potential of kynurenic acid (KYNA) and quercetin, two naturally derived compounds, to modulate the immune response of MSCs through the regulation of AHR signaling under hyperglycemic conditions. We assessed the immunophenotyping and differentiation capacity of cultured human umbilical cord mesenchymal stem cells (hUC-MSCs) in a high-glucose medium and quantified the mRNA expression rate of *AHR*, *CYP1A1*, *CYP1B1*, and *IL-6* using real time PCR. Our study is the first to reveal that KYNA and quercetin enhance mRNA expression levels of *AHR* and *CYP1B1*, while reducing *IL-6* expression in hUC-MSCs, suggesting their potential as immunomodulators. These findings highlight the compounds' promise as drug candidates for immune-mediated diseases through stem cell therapy, particularly due to their modulation of AHR.

## 1. Introduction

Hyperglycemia is a well-known metabolic derangement, defined as a blood glucose concentration greater than 140 mg/dL (7.8 mmol/L). Hyperglycemia is the most prominent sign that characterizes diabetes [1–3], which is related to at least 463 million adults worldwide, making up about 9.3% of the global adult population [4]. Hyperglycemia can cause injury to a large number of organs and tissues [5], leading to acute and chronic complications and threatening several health concerns, such as diabetic ketoacidosis, vacular diseases, or impairment of cognitive function [6, 7]. Many cells in the human body are unable to adapt to high glucose concentrations, and they equilibrate their intracellular glucose level to the extracellular concentrations, and therefore, are more susceptible to the effect of hyperglycemia [8–10].

MSCs are multipotent progenitor cells distinguished by their capacity for self-renewal and their ability to differentiate into multiple cell lineages. These cells are readily obtainable and can be isolated and expanded ex vivo from a wide range of tissues within the body. A key attribute of MSCs is their robust anti-inflammatory and immunosuppressive capabilities [11–17], which lead to several applications in immune-related diseases [18]. These cells have been shown to modulate the functions of various immune cells, including T cells, B cells, dendritic cells, and macrophages. MSCs inhibit T-cell proliferation [16, 17, 19], and impact T cell differentiation, particularly by suppressing the development of Th17 and Tc17 cells [20–22]. However, several studies reported that MSCs in hyperglycemic conditions showed decreased immunomodulatory properties [23, 24]. Therefore, the development of a novel approach to recover the immunomodulation potential of MSC is necessary.

The aryl hydrocarbon receptor (AHR) is a ligand-activated receptor known for mediating the toxic effects of environmental pollutants, including 2,3,7,8-tetrachlorodibenzo-p-dioxin (TCDD) [25]. Upon binding to TCDD, AHR translocates from the cytosol to the nucleus, where it alters the transcription of target genes, such as those encoding cytochrome P450 isoforms CYP1A1 and CYP1B1, subsequently inducing a range of immunotoxicological responses [26, 27]. Although AHR is primarily associated with adaptive responses to chemical exposure, emerging evidence indicates its involvement in immune regulation through various pathways [28]. It has been established that MSCs express AHR, and that exposure to cockroach allergen can activate this receptor, leading to upregulated expression of downstream genes like CYP1A1 and CYP1B1 [29]. Additionally, the modulation of MSCs therapeutic potential by AHR activation has been observed; for instance, stimulation of MSCs with a natural AHR agonist was shown to enhance their immunosuppressive properties, particularly by reducing the expression of IL-6 [30]. These observations suggest that the immunomodulatory functions of MSCs may be significantly influenced by AHR activity, indicating that AHR activation may be crucial for MSCs to exert their immunomodulatory effects.

**Figure 1:**
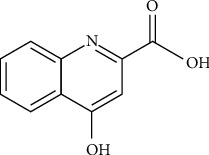
Chemical structure of kynurenic acid.

KYNA (Figure 1) is a metabolite of tryptophan produced by astrocytes, recognized for its neuroinhibitory activity. It functions as an antagonist to ionotropic glutamate receptors, with a particularly high affinity for N-methyl-D-aspartate receptors [31]. KYNA is also generated through the enzymatic action of indoleamine 2,3-dioxygenase (IDO) and has been reported to activate the AHR. This activation allows AHR to bind directly to the TSG-6 promoter, thereby promoting TSG-6 expression [32]. Furthermore, pretreating MSCs with KYNA has been demonstrated to augment their therapeutic effects in a murine model of acute lung injury [33].

**Figure 2:**
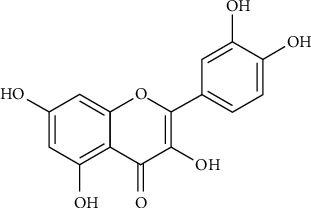
Chemical structure of quercetin.

Quercetin (Figure 2) is a polyphenolic compound classified within the flavonoid family and is abundantly present in dietary sources such as apples, berries, grapes, onions, and tea [34]. It has long been recognized for its potential in preventing and treating various chronic conditions in humans, including allergic airway diseases, organ fibrosis, cardiovascular and neurological disorders, diabetes, and cancer [35]. The anti-inflammatory properties of quercetin have been extensively studied, with evidence suggesting its involvement in a range of pharmacological activities [36–40]. In particular, quercetin has been demonstrated to induce MSCs to secrete anti-inflammatory mediators, such as the enzyme IDO, nitric oxide, and IL-6, when cocultured with peripheral blood mononuclear cells that have been pretreated with tumor necrosis factor-alpha (TNF-α) and interferon gamma (IFN-γ) [41, 42].

In this work, we examined the anti-inflammatory properties of KYNA and quercetin at various concentrations on hUC-MSCs in a hyperglycemic milieu. Throughout the study, high-glucose Dulbecco's Modified Eagle Medium (DMEM) was used as the culture condition for hUC-MSCs. Then, the surface phenotype and differentiation potential of the cultured hUC-MSCs were assessed. Additionally, reverse transcription polymerase chain reaction (RT-PCR) was employed to assess the mRNA expression levels of AHR, CYP1A1, CYP1B1, and IL-6 in order to evaluate the immunomodulatory activity of KYNA and quercetin.

## 2. Materials and Methods

### 2.1. hUC-MSCs Isolation and Culture

hUC-MSCs were isolated and cultured following the previous publication [43]. All experiments were conducted with approval from the Ethical Committee of the Vietnam Academy of Science and Technology.

The hUC-MSCs were seeded in high-glucose DMEM (Capricorn Scientific, Germany), along with 10% fetal bovine serum (FBS, Capricorn Scientific, Germany) and 1% penicillin–streptomycin (Gibco, USA). Initial culturing involved seeding hUC-MSCs in T175 flasks (Eppendorf, Germany) and maintaining them in an incubator set at 37°C with a 5% CO_2_ atmosphere. To ensure optimal cell growth and viability, the culture medium was exchanged every 2–3 days. After allowing the cell cultures to reach a confluency rate of 70%–80%, the detachment was achieved using a trypsin-EDTA solution (Gibco, USA) and reseeded at the required densities for further expansion. For all experiments, hUC-MSCs at passages 3–5 (P3 to P5) were utilized.

### 2.2. Immunophenotyping Characterization of Cultured hUC-MSCs

Cell surface markers were characterized according to methods described in previous publications [44]. Briefly, hUC-MSCs were rinsed with phosphate-buffered saline (PBS), then centrifuged, harvested, and resuspended in PBS. The harvested cells were grown using 100 μL of PBS containing an antibody cocktail comprising CD45-APC (BioLegend, USA), CD90-FITC (BioLegend, USA), and CD105-APC-A750 (BioLegend, USA) for 30 min at room temperature. Following incubation, the hUC-MSCs were washed with PBS as well as analyzed using the MACSQuant VYB flow cytometer (Miltenyi Biotec, USA). Data were processed and evaluated with MACSQuantify Software (Miltenyi Biotec, USA).

### 2.3. Differentiation Assay of Cultured hUC-MSCs

For osteogenic differentiation, hUC-MSCs were cultured in OsteoDiff medium (StemMACS, USA) for 14 days, with the medium replaced every 4–5 days. After the differentiation period, washed the cells twice using PBS, then fixed the cells for 30 min with 4% formaldehyde. Subsequently, using Alizarin red (Sigma Aldrich, USA), the cells were stained for 5 min to evaluate calcium deposition.

For adipogenic differentiation, hUC-MSCs were incubated in AdipoDiff medium (StemMACS, USA) for 14 days, with medium changes every 4–5 days. Following differentiation, the harvested cells were washed twice by using PBS, then perform fixing for 30 min in 4% formaldehyde. Then, stain the cells with Oil Red O (Sigma Aldrich, USA) for 30 min to visualize lipid vacuole formation.

### 2.4. Real-Time PCR Analysis

Once the cells reached an appropriate density, they were pretreated with varying concentrations of KYNA (0, 10, 50, 100, and 200 μM) for 8 h, or quercetin (0, 10, 50, and 100 μM) for 16 h to assess the mRNA expression levels of *AHR*, *CYP1A1*, and *CYP1B1*. To investigate *IL-6* expression, KYNA was incubated with hUC-MSCs for 16 h, while quercetin was incubated for 24 h.

For the analysis of quantitative real-time PCR, cells were harvested at each time point, centrifuged to remove supernatant, and TRIzol Reagent (Invitrogen, USA) was implemented for total RNA isolation. The synthesis of DNA via reverse transcription was conducted using the RevertAid First Strand cDNA Synthesis Kit (Thermo Fisher Scientific, USA). Then, quantitative PCR reactions were prepared with Power Green PCR Master Mix (Applied Biosystems, USA) following the manufacturer's guidelines to express levels of five genes namely, *AHR*, *CYP1A1*, *CYP1B1*, and *IL-6* with β*-actin* as the internal control. The primers' sequences employed in this study were: *AHR:* 5′-TGGTCTCCCCCAGACAGTAG-3′ (forward), 5′- TTCATTGCCAGAAAACCAGA-3′ (reverse); *CYP1A1:* 5′-TAGACACTGATCTGGCTGCAG-3′ (forward), 5′-GGGAAGGCTCCATCAGCATC-3′ (reverse); *CYP1B1:* 5′-GCTGCAGTGGCTGCTCCT-3′ (forward), 5′-CCCACGACCTGATCCAATTCT-3′ (reverse); *IL-6:* 5′-TGAACTCCTTCTCCACAAGCG-3′ (forward), 5′-TGGTGTTGCCTGCTGTCCCT-3′ (reverse); *B-Actin:* 5′-TGGCCGAGGACTTTGATTG-3′ (forward), 5′-TGTGTGGACTTGGGAGAGGA-3′ (reverse).

### 2.5. Statistical Analysis

The data were expressed as the mean ± standard deviation (SD). To assess the data, one-way ANOVA was performed, with statistical significance defined as *p*  < 0.05.

## 3. Results

### 3.1. Characterization of Cultured hUC-MSCs

**Figure 3:**
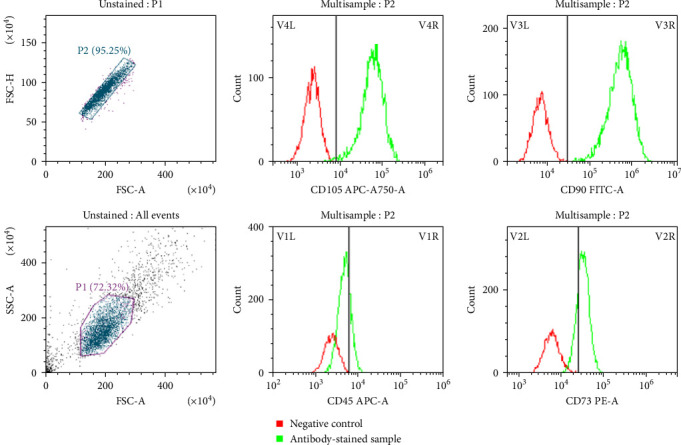
Phenotypic characterization of hUC-MSCs by flow cytometric analysis.

**Figure 4:**
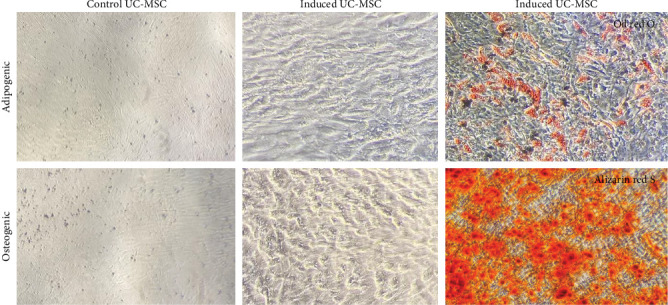
Morphology and differentiation capacity of hUC-MSCs.

Characterization of the hUC-MSCs cultured in high-glucose medium was evaluated by immunophenotyping and differentiation. The results indicated that the cultured hUC-MSCs expressed CD105, CD90, and CD73 and lacked expression of CD45, corresponding to the nature of mesenchymal stem cells (Figure 3). Additionally, the cultured hUC-MSCs exhibited the capacity to differentiate into osteocytes and adipocytes (Figure 4). These results collectively confirm the identity and functional potential of the hUC-MSC.

### 3.2. Immunoregulation of KYNA in hUC-MSCs

cDNA from the hUC-MSCs samples treated with different concentrations of KYNA at different time points, followed by RT-PCR analysis. β-actin was used as the reference gene for normalization, with statistical significance set at *p*  < 0.05.

**Figure 5:**
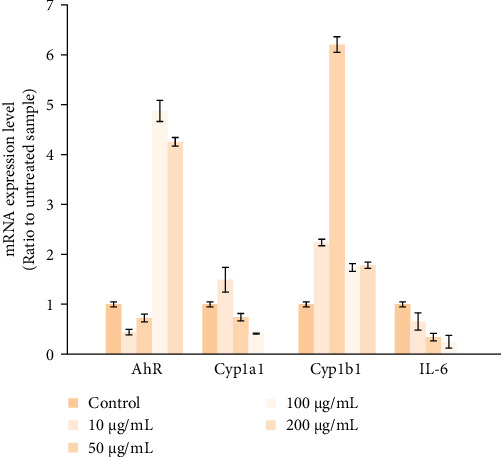
Gene expression levels in UC-MSCs treated with KYNA. cDNA from the hUC-MSCs samples treated with different concentrations of KYNA in different time points, followed by RT-PCR analysis. β-Actin was used as the reference gene for normalization, with statistical significance set at *p* < 0.05.

The immunoregulatory effect of KYNA on *AHR*, *CYP1A1*, *CYP1B1*, and *IL-6′s* mRNA expression levels was evaluated by RT-PCR in hUC-MSCs cultured in hyperglycemic conditions. After the incubation with KYNA, when the concentration of KYNA increased to higher 50 μg/μL, the mRNA expression level of *AHR* significantly rose to 129%, while the mRNA content of *CYP1B1* rose to 629% (Figure 5). By contrast, the mRNA expression of both *CYP1A1* and *IL-6* reduced in a dose-dependent manner.

### 3.3. Immunoregulation of Quercetin in hUC-MSCs

cDNA from the hUC-MSCs samples treated with different concentrations of quercetin at different time points, followed by RT-PCR analysis. β-actin was used as the reference gene for normalization, with statistical significance set at *p*  < 0.05.

**Figure 6:**
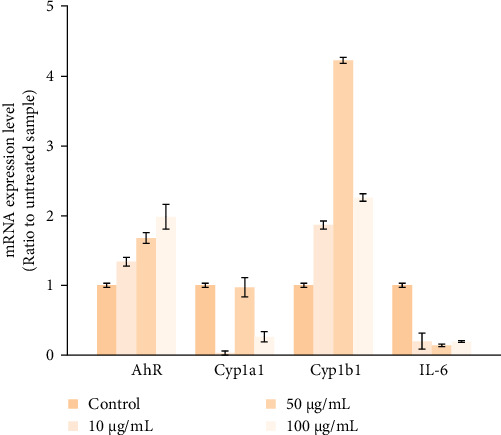
Gene expression levels in UC-MSCs treated with quercetin. cDNA from the hUC-MSCs samples treated with different concentrations of quercetin in different time points, followed by RT-PCR analysis. β-Actin was used as the reference gene for normalization, with statistical significance set at *p* < 0.05.

The inhibitory effect of quercetin on *AHR*, *CYP1A1*, *CYP1B1*, and *IL-6* mRNA expression levels was evaluated by real time PCR in cultured hUC-MSCs. As shown in Figure 6, quercetin significantly induced the production of *AHR* mRNA in a dose-dependent manner to over 200%, while the mRNA levels of *CYP1B1* even rose to 428%. By contrast, the compound remarkably inhibited the *IL-6* and *CYP1A1* mRNA expression levels.

## 4. Discussion

In this study, hUC-MSCs were successfully isolated and expanded, preserving their characteristic surface markers and their capability to differentiate into both adipocytes and osteocytes after several passages cultured in hyperglycemic milieu. Generally, the data indicated that KYNA and quercetin both stimulated the mRNA production of the *AHR* and *CYP1B1*. In contrast, the inhibition of these compounds on the mRNA expression levels of *CYP1A1* and interleukin-6 was observed.

Currently, MSCs are characterized by the positive expression of CD105, CD90, and CD73, and the absence of HLA-DR, CD14, CD34, or CD79a, CD11b, or CD19, and CD45 markers. While MSCs from different sources often present similar immunophenotypic profiles according to these markers, studies have noted variations in the expression of additional markers [45]. For instance, CD45 is recognized as a negative marker for MSCs [44], which is typically expressed in leukocytes [46]. In contrast, CD90, CD105, and CD73 are established as positive markers for MSCs [47]. Moreover, CD146 is expressed by UC-MSCs but is absent in MSCs from adipose tissue (AT) or bone marrow (BM). Furthermore, CD133 is frequently found in BM- and AT-MSCs, while CD34 expression is exclusive to AT-MSCs [48]. Additionally, the capability of MSCs to differentiate into osteocytes and adipocytes is a critical characteristic for defining these cells [49]. In this study, no substantial changes were observed in the proportions of cells negative for CD45 and positive for CD105 and CD90, nor their differentiation capacity, following culture in DMEM supplemented with 10% FBS. This suggests that the culture conditions did not impact the expression levels of cell surface markers or the biological functions of the MSCs.

One of the most notable features of UC-MSCs is their biological capability to modulate immune responses. Despite not fully comprehending the precise mechanism of action, both direct cell-to-cell interactions and the release of soluble factors are crucial components of the immunomodulatory effects exerted by UC-MSCs. These immunoregulatory activities are facilitated by the release of antigen-presenting cells and inflammatory cytokines from T cells, including interleukin-1 α, interleukin-1 β, IFN-γ, and TNF-α [45, 50]. UC-MSCs are particularly noted for their ability to inhibit effector T cell functions [51]. They achieve this by activating cell cycle arrest alongside apoptosis in T cells through the release of IDO [52], PGE-2, and TGF-β1 [53, 54]. Additionally, UC-MSCs attenuate the proliferation as well as activation of T cells by modulating their phenotypes as well as increasing the proportion of Tregs. This immunosuppressive effect is controlled by the secretion of anti-inflammatory cytokines, including IL-10, PGE-2, and TGF-β1, and a concomitant reduction in the expression level of IFN-γ [45, 55]. In the early phases of the immune response, UC-MSCs inhibit dendritic cell maturation and differentiation, promoting the polarization of the monocytes towards an IL-10-expressing phenotype through the secretion of IL-6 and HGF [56]. They also influence macrophage polarization by suppressing M1 macrophage activation and encouraging M2 macrophage differentiation through TNF-stimulated gene-6 (TSG-6) and TNF-α-mediated activation of COX-2 [57]. UC-MSCs induce M2 macrophage polarization via the release of monocyte chemoattractant protein-1 as well as IL-6 [58]. Additionally, UC-MSCs produce PGE-2 and activin-A, which inhibit IFN-γ expression by natural killer (NK) cells [59]. The NK-cell-cytolytic function is further diminished by the UC-MSCs derived immunosuppressive isoform of human leukocyte antigen-G6 secreted [51]. The impact of UC-MSCs on B cells remains less well-characterized. Some studies found that UC-MSCs could hinder proliferation, antibody secretion of B cells, and differentiation [60], whereas others reported no significant effects [61]. The mechanisms of action described are predominantly based on in vitro studies, with limited *i*n vivo research available on the effects of UC-MSCs on immune cell populations. In vitro *a*ssays revealed that UC-MSCs elicit minimal immune responses of allogeneic T cells, showing the low immunogenic potential. This characteristic is likely due to the substantial secretion of immunoregulatory elements, such as TGF-β1 or IL-10. Additionally, UC-MSCs display low levels of HLA class I expression and lack the expression of HLA-DR as well as costimulatory molecules, such as CD40, CD80, and CD86 markers [51, 54, 62]. Furthermore, UC-MSCs are unique in their secretion of the IFN-α, unlike BM-MSCs. In response to IL-1β, UC-MSCs exhibit increased levels of TGF-β1, PGE-2, TSG-6, and IDO [63, 64]. Importantly, UC-MSCs do not induce *HLA-DR* expression upon stimulation with IFN-γ, distinguishing them from BM-MSCs and underscoring their lower immunogenic profile, which may be advantageous for allogeneic use in the context of inflammatory diseases [51].

AHR has emerged as a significant regulator in various cellular functions, including its influence on the activity of nuclear factor kappa-light-chain-enhancer of activated B cells (NF-*κ*B) [65, 66]. Research utilizing *AHR* knockout mice has shown an elevated presence of pre-B cells in comparison with wild-type counterparts, indicating that *AHR* expression is likely induced by B cell activation [67, 68]. Additionally, *AHR*-deficient mice exhibit increased vulnerability to septic shock, a condition potentially mediated through NF-*κ*B signaling pathways [66]. AHR's role extends to the modulation of immune responses. The prototypical AHR agonist, TCDD, a known environmental toxicant and carcinogen, has been the subject of extensive research based on its effects on inflammation. Upon binding to TCDD, AHR moves from the cytosol to the nucleus, where it alters the transcription of target genes, such as those encoding cytochrome P450 isoforms *CYP1B1* and *CYP1A1*, subsequently inducing a range of immunotoxicology responses [26, 27]. Evidence indicates that TCDD-mediated activation of AHR leads to a reduction in inflammatory responses by altering T cell subsets and cytokine profiles. For instance, in a pertussis toxin-induced murine model, treatment of TCDD resulted in the reduction of proinflammatory cytokines, along with an increase in Tregs as well as anti-inflammatory ones [69]. Furthermore, AHR activation under induction of TCDD has been implicated in modulating the activity or function of Th17 cells, dendritic cells, and macrophages [70–73]. In addition to exogenous ligands, endogenous AHR activators, such as KYN derived from tryptophan metabolism, have also been identified. AHR activation induced by KYN can promote the differentiation of T cells towards FoxP3 (+) Tregs rather than Th17 [74]. AHR traditionally controls the production of phase 1 metabolizing enzymes belonging to the cytochrome P450 superfamily, which are involved in the oxidation and excretion of xenobiotics. KYN has been shown to modulate various immunological responses in MSCs. KYN-stimulated MSCs exhibit reduced levels of IL-6, increased expression of cyclooxygenase 2, and suppression of Th17 cell differentiation [30]. Collectively, these findings imply that the immunosuppressive effects mediated by MSCs may be contingent upon the activation of AHR.

KYNA is a metabolite of tryptophan produced by astrocytes, recognized for its neuroinhibitory activity. It functions as an antagonist to ionotropic glutamate receptors, with a particularly high affinity for N-methyl-D-aspartate receptors [31]. KYNA is also generated through the enzymatic action of IDO and has been reported to activate the AHR. This activation allows AHR to bind directly to the TSG-6 promoter, thereby promoting TSG-6 expression [32]. Furthermore, pretreating MSCs with KYNA has been demonstrated to augment their therapeutic effects on acute lung injury in mouse models [33]. KYNA can stimulate the expression of TSG-6 in MSCs through the IDO pathway [75]. The genetic deletion of IDO or pharmacological suppression of IDO activity leads to reduced TSG-6 expression and diminishes the anti-inflammatory effects of MSCs [33]. Meanwhile, quercetin has been documented to influence immune responses and inflammation through its interactions with various enzymes, intracellular signaling kinases, phosphatases, and membrane proteins essential for cellular functions [76]. It has been identified as an antagonist of the AHR, inhibiting receptor activation induced by TCDD [77]. Quercetin has been shown to induce a transient increase in mRNA expression levels of *CYP1A1* and ethoxyresorufin-O-deethylase activity in MCF-7 cells [78] as well as to enhance *CYP1A1* expression and facilitate AHR nuclear translocation in Caco-2 cells [79]. Furthermore, quercetin has been reported to modulate MSC inflammation by reducing the production of TNF-α and the secretion of various inflammatory cytokines in MSCs derived from patients affected by type 2 diabetes and impaired glucose tolerance [80].

Hyperglycemia is known to play a pivotal role in inflammation. Sustained high glucose elevates mitochondrial reactive oxygen species, activates NF-κB–dependent signaling, and boosts secretion of proinflammatory cytokines such as TNF-α and IL-6, thereby propagating oxidative stress and inducing tissue damage [81–83]. These cytokine-rich conditions diminish key immunomodulatory mechanisms, such as indoleamine-2,3-dioxygenase, prostaglandin E_2_, and TGF-β, down-regulates migratory CXCR4/SDF-1 signaling and diminish MSC survival in vivo, collectively eroding their immunomodulatory efficacy [84–87]. Consequently, these mechanism are driven by hyperglycemia may not only exacerbate tissue damage but also markedly diminish the functional potential of both endogenous and transplanted MSCs, highlighting the necessity of control and immunomodulation for successful MSC-based interventions in patients.

As mentioned above, it is previously reported that ligand-activated *AhR* translocates to the nucleus and co-activates xenobiotic response elements, inducing *CYP1A1* and *CYP1B1* transcription in tandem. Some previous studies reported that higher AhR levels lead to increased expression of both *CYP1A1* and *CYP1B1* [27, 88]. In our study, *CYP1B1* was upregulated without a commensurate rise in *AhR* mRNA, and their dose-response trends were atypical. It was previously demonstrated that *CYP1B1* expression can be constitutively high and inducible even in *AhR*-knockout cells [89, 90]. Additionally, chronic hyperglycemia is known to generate elevated ROS, and together these factors can modulate cytochrome P450 expression. High-glucose conditions drive oxidative stress that impacts cellular gene expression programs of some specific CYP enzymes. On the other hand, inflammation secondary to hyperglycemia may downregulate some drug-metabolizing CYPs, while upregulating others like *CYP1B1*, indicating a complex regulation by metabolic stress and ROS [90–92]. Thus, our results may imply that *CYP1B1* might be regulated through AhR-independent mechanisms, especially under hyperglycemic stress.

On the other hand, it was reported that in a murine model, a high dose of quercetin significantly decreased *CYP1A1* expression in the liver, even though *AhR* expression was not affected [93]. By contrast, in human HepG2 cells, quercetin at concentrations above 10 µM induced levels of *CYP1A1* mRNA and activity, whereas at lower concentrations, no significant induction was observed [94]. In our study, quercetin treatment at high doses did not reduce CYP1A1 mRNA levels as observed at 10 µg/mL. This might suggest that quercetin's effects on CYP1A1 expression can vary depending on the experimental conditions and the concentration used. This compound could act as an antioxidant at lower concentrations but can exhibit prooxidant or cytotoxic properties at high concentrations, which is a known inducer of CYP1A1 expression [95, 96]. While such nonlinear dose responses are well recognized with many bioactive compounds, further study is necessary to clarify these findings.

## 5. Conclusion

For the first time, our study revealed that under hyperglycemic stress, both KYNA and quercetin enhanced the mRNA expression levels of *AHR* and *CYP1B1*, as well as reduced the expression of *CYP1A1* and *IL-6* in hUC-MSCs. Although further investigation is required to elucidate the pharmaceutical mechanisms of KYNA and quercetin in hUC-MSCs under hyperglycemic conditions, these highlighted that these compounds might act as immunomodulators, contributing to the suppression of inflammatory responses of MSCs. Collectively, these two natural compounds could be promising drug candidates for therapeutic intervention in immune-mediated diseases through stem cell therapy, particularly due to their involvement in AHR modulation. In the future, we would conduct further research to comprehensively assess their efficacy and validate their mechanisms of action in both in vivo and in vitro settings.

## Data Availability

The data that support the findings of this study are available from the corresponding author upon reasonable request.

## References

[1] Umpierrez G. E., Hellman R., Korytkowski M. T. (2012). Management of Hyperglycemia in Hospitalized Patients in Non-Critical Care Setting: An Endocrine Society Clinical Practice Guideline. *The Journal of Clinical Endocrinology & Metabolism*.

[2] Moghissi E. S., Korytkowski M. T., DiNardo M. (2009). American Association of Clinical Endocrinologists and American Diabetes Association Consensus Statement on Inpatient Glycemic Control. *Diabetes Care*.

[3] Dhatariya K., James J., Kong M.-F., Berrington R., the Joint British Diabetes Society (JBDS) for Inpatient Care Group and Guidelines Writing Group (2020). Diabetes at the Front Door. A Guideline for Dealing With Glucose Related Emergencies at the Time of Acute Hospital Admission From the Joint British Diabetes Society (JBDS) for Inpatient Care Group. *Diabetic Medicine*.

[4] Yapislar H., Gurler E. B. (2024). Management of Microcomplications of Diabetes Mellitus: Challenges, Current Trends, and Future Perspectives in Treatment. *Biomedicines*.

[5] Giugliano D., Ceriello A., Esposito K. (2008). Glucose Metabolism and Hyperglycemia. *The American Journal of Clinical Nutrition*.

[6] Amiel S. A. (2021). The Consequences of Hypoglycaemia. *Diabetologia*.

[7] Marcovecchio M. L. (2017). Complications of Acute and Chronic Hyperglycemia. *US Endocrinology*.

[8] Shrayyef M. Z., Gerich J. E., Poretsky L. (2010). Normal Glucose Homeostasis. *Principles of Diabetes Mellitus*.

[9] Szablewski L. (2011). *Glucose Homeostasis and Insulin Resistance*.

[10] Brownlee M. (2005). The Pathobiology of Diabetic Complications: A Unifying Mechanism. *Diabetes*.

[11] Le Blanc K., Tammik C., Rosendahl K., Zetterberg E., Ringdén O. (2003). HLA Expression and Immunologic Properties of Differentiated and Undifferentiated Mesenchymal Stem Cells. *Experimental Hematology*.

[12] Le Blanc K., Mougiakakos D. (2012). Multipotent Mesenchymal Stromal Cells and the Innate Immune System. *Nature Reviews Immunology*.

[13] Rasmusson I., Uhlin M., Blanc K. Le, Levitsky V. (2007). Mesenchymal Stem Cells Fail to Trigger Effector Functions of Cytotoxic T Lymphocytes. *Journal of Leukocyte Biology*.

[14] Rasmusson I., Ringdén O., Sundberg B., Blanc K. Le (2005). Mesenchymal Stem Cells Inhibit Lymphocyte Proliferation by Mitogens and Alloantigens by Different Mechanisms. *Experimental Cell Research*.

[15] Sundin M., Barrett A. J., Ringdén O. (2009). HSCT Recipients Have Specific Tolerance to MSC but Not to the MSC Donor. *Journal of Immunotherapy*.

[16] Aggarwal S., Pittenger M. F. (2005). Human Mesenchymal Stem Cells Modulate Allogeneic Immune Cell Responses. *Blood*.

[17] Glennie S. J., Soeiro I., Dyson J., Lam E., Dazzi F. (2005). Mesenchymal Stem Cells Induce Division Arrest Anergy of Activated T Cells. *Biology of Blood and Marrow Transplantation*.

[18] Mallis P., Michalopoulos E., Chatzistamatiou T., Giokas C. S. (2021). Interplay Between Mesenchymal Stromal Cells and Immune System: Clinical Applications in Immune-Related Diseases. *Exploration of Immunology*.

[19] English K., Ryan J. M., Tobin L., Murphy M. J., Barry F. P., Mahon B. P. (2009). Cell Contact, Prostaglandin E2 and Transforming Growth Factor Beta 1 Play Non-Redundant Roles in Human Mesenchymal Stem Cell Induction of CD4+ CD25Highforkhead Box P3+ Regulatory T Cells. *Clinical and Experimental Immunology*.

[20] Glenn J. D., Smith M. D., Calabresi P. A., Whartenby K. A. (2014). Mesenchymal Stem Cells Differentially Modulate Effector CD8+ T Cell Subsets and Exacerbate Experimental Autoimmune Encephalomyelitis. *Stem Cells*.

[21] Ghannam S., Pène J., Torcy-Moquet G., Jorgensen C., Yssel H. (2010). Mesenchymal Stem Cells Inhibit Human Th17 Cell Differentiation and Function and Induce a T Regulatory Cell Phenotype. *The Journal of Immunology*.

[22] Luz-Crawford P., Noël D., Fernandez X. (2012). Mesenchymal Stem Cells Repress Th17 Molecular Program Through the PD-1 Pathway. *PLoS ONE*.

[23] Montanucci P., Pescara T., Pennoni I. (2018). Functional Profiles of Human Umbilical Cord-Derived Adult Mesenchymal Stem Cells in Obese/Diabetic Versus Healthy Women. *Current Diabetes Reviews*.

[24] Mateen M. A., Alaagib N., Haider K. H. (2024). High Glucose Microenvironment and Human Mesenchymal Stem Cell Behavior. *World Journal of Stem Cells*.

[25] Denison M. S., Nagy S. R. (2003). Activation of the Aryl Hydrocarbon Receptor by Structurally Diverse Exogenous and Endogenous Chemicals. *Annual Review of Pharmacology and Toxicology*.

[26] Gonzalez F. J., Fernandez-Salguero P. (1998). The Aryl Hydrocarbon Receptor: Studies Using the AHR-Null Mice. *Drug Metabolism and Disposition*.

[27] Stockinger B., Meglio P. D., Gialitakis M., Duarte J. H. (2014). The Aryl Hydrocarbon Receptor: Multitasking in the Immune System. *Annual Review of Immunology*.

[28] Quintana F. J., Sherr D. H. (2013). Aryl Hydrocarbon Receptor Control of Adaptive Immunity. *Pharmacological Reviews*.

[29] Xu T., Zhou Y., Qiu L. (2015). Aryl Hydrocarbon Receptor Protects Lungs From Cockroach Allergen-Induced Inflammation by Modulating Mesenchymal Stem Cells. *The Journal of Immunology*.

[30] Hinden L., Shainer R., Almogi-Hazan O., Or R. (2015). Ex Vivo Induced Regulatory Human/Murine Mesenchymal Stem Cells as Immune Modulators. *Stem Cells*.

[31] Stone T. W. (2000). Development and Therapeutic Potential of Kynurenic Acid and Kynurenine Derivatives for Neuroprotection. *Trends in Pharmacological Sciences*.

[32] DiNatale B. C., Murray I. A., Schroeder J. C. (2010). Kynurenic Acid Is a Potent Endogenous Aryl Hydrocarbon Receptor Ligand That Synergistically Induces Interleukin-6 in the Presence of Inflammatory Signaling. *Toxicological Sciences*.

[33] Wang G., Cao K., Liu K. (2018). Kynurenic Acid, an IDO Metabolite, Controls TSG-6-Mediated Immunosuppression of Human Mesenchymal Stem Cells. *Cell Death & Differentiation*.

[34] Gormaz J. G., Quintremil S., Rodrigo R. (2015). Cardiovascular Disease: A Target for the Pharmacological Effects of Quercetin. *Current Topics in Medicinal Chemistry*.

[35] Miles S. L., McFarland M., Niles R. M. (2014). Molecular and Physiological Actions of Quercetin: Need for Clinical Trials to Assess its Benefits in Human Disease. *Nutrition Reviews*.

[36] Min Y.-D., Choi C.-H., Bark H. (2007). Quercetin Inhibits Expression of Inflammatory Cytokines Through Attenuation of NF-*κ*B and p38 MAPK in HMC-1 Human Mast Cell Line. *Inflammation Research*.

[37] Rogerio A., Kanashiro A., Fontanari C. (2007). Anti-Inflammatory Activity of Quercetin and Isoquercitrin in Experimental Murine Allergic Asthma. *Inflammation Research*.

[38] Chuang C.-C., Martinez K., Xie G. (2010). Quercetin is Equally or More Effective Than Resveratrol in Attenuating Tumor Necrosis Factor-*α*–mediated Inflammation and Insulin Resistance in Primary Human Adipocytes. *The American Journal of Clinical Nutrition*.

[39] Ortega M. G., Saragusti A. C., Cabrera J. L., Chiabrando G. A. (2010). Quercetin Tetraacetyl Derivative Inhibits LPS-Induced Nitric Oxide Synthase (iNOS) Expression in J774A.1 Cells. *Archives of Biochemistry and Biophysics*.

[40] Ishizawa K., Izawa-Ishizawa Y., Ohnishi S. (2009). Quercetin Glucuronide Inhibits Cell Migration and Proliferation by Platelet-Derived Growth Factor in Vascular Smooth Muscle Cells. *Journal of Pharmacological Sciences*.

[41] Chen G., Ye Y., Cheng M. (2020). Quercetin Combined With Human Umbilical Cord Mesenchymal Stem Cells Regulated Tumour Necrosis Factor-*α*/Interferon-*γ*-Stimulated Peripheral Blood Mononuclear Cells via Activation of Toll-Like Receptor 3 Signalling. *Frontiers in Pharmacology*.

[42] Liu J., Li X., Yue Y., Li J., He T., He Y. (2005). The Inhibitory Effect of Quercetin on IL-6 Production by LPS-Stimulated Neutrophils. *Cellular & Molecular Immunology*.

[43] Lu L.-L., Liu Y.-J., Yang S.-G. (2006). Isolation and Characterization of Human Umbilical Cord Mesenchymal Stem Cells With Hematopoiesis-Supportive Function and Other Potentials. *Haematologica*.

[44] Dominici M., Blanc K. Le, Mueller I. (2006). Minimal Criteria for Defining Multipotent Mesenchymal Stromal Cells. The International Society for Cellular Therapy Position Statement.. *Cytotherapy*.

[45] Mebarki M., Abadie C., Larghero J., Cras A. (2021). Human Umbilical Cord-Derived Mesenchymal Stem/Stromal Cells: A Promising Candidate for the Development of Advanced Therapy Medicinal Products. *Stem Cell Research & Therapy*.

[46] Rheinländer A., Schraven B., Bommhardt U. (2018). CD45 in Human Physiology and Clinical Medicine. *Immunology Letters*.

[47] Ramos L., L. I. Sánchez-Abarca T., Muntión S. (2016). MSC Surface Markers (CD44, CD73, and CD90) Can Identify Human MSC-Derived Extracellular Vesicles by Conventional Flow Cytometry. *Cell Communication and Signaling*.

[48] Petrenko Y., Vackova I., Kekulova K. (2020). A Comparative Analysis of Multipotent Mesenchymal Stromal Cells Derived From Different Sources, With a Focus on Neuroregenerative Potential. *Scientific Reports*.

[49] Halim A., Ariyanti A. D., Luo Q., Song G. (2020). Recent Progress in Engineering Mesenchymal Stem Cell Differentiation. *Stem Cell Reviews and Reports*.

[50] De Witte S. F., Merino A. M., Franquesa M. (2017). Cytokine Treatment Optimises the Immunotherapeutic Effects of Umbilical Cord-Derived MSC for Treatment of Inflammatory Liver Disease. *Stem Cell Research & Therapy*.

[51] Weiss M. L., Anderson C., Medicetty S. (2008). Immune Properties of Human Umbilical Cord Wharton’s Jelly-Derived Cells. *Stem Cells*.

[52] Gutta S., Shenoy J., Kamath S. P. (2019). Light Emitting Diode (LED) Phototherapy Versus Conventional Phototherapy in Neonatal Hyperbilirubinemia: A Single Blinded Randomized Control Trial From Coastal India. *BioMed Research International*.

[53] Najar M., Raicevic G., Boufker H. I. (2010). Mesenchymal Stromal Cells Use PGE2 to Modulate Activation and Proliferation of Lymphocyte Subsets: Combined Comparison of Adipose Tissue, Wharton’s Jelly and Bone Marrow Sources. *Cellular Immunology*.

[54] Zhou C., Yang B., Tian Y. (2011). Immunomodulatory Effect of Human Umbilical Cord Wharton’s Jelly-Derived Mesenchymal Stem Cells on Lymphocytes. *Cellular Immunology*.

[55] Yang H., Sun J., Wang F., Li Y., Bi J., Qu T. (2016). Umbilical Cord-Derived Mesenchymal Stem Cells Reversed the Suppressive Deficiency of T Regulatory Cells From Peripheral Blood of Patients With Multiple Sclerosis in a Co-Culture-a Preliminary Study. *Oncotarget*.

[56] Deng Y., Zhang Y., Ye L. (2016). Umbilical Cord-Derived Mesenchymal Stem Cells Instruct Monocytes Towards an IL10-Producing Phenotype by Secreting IL6 and HGF. *Scientific Reports*.

[57] Shin T.-H., Kim H.-S., Kang T.-W. (2016). Human Umbilical Cord Blood-Stem Cells Direct Macrophage Polarization and Block Inflammasome Activation to Alleviate Rheumatoid Arthritis. *Cell Death & Disease*.

[58] Yin Y., Hao H., Cheng Y. (2018). Human Umbilical Cord-Derived Mesenchymal Stem Cells Direct Macrophage Polarization to Alleviate Pancreatic Islets Dysfunction in Type 2 Diabetic Mice. *Cell Death & Disease*.

[59] Chatterjee D., Marquardt N., Tufa D. M. (2014). Human Umbilical Cord-Derived Mesenchymal Stem Cells Utilize Activin-A to Suppress Interferon-*γ* Production by Natural Killer Cells. *Frontiers in Immunology*.

[60] Che N., Li X., Zhou S. (2012). Umbilical Cord Mesenchymal Stem Cells Suppress B-Cell Proliferation and Differentiation. *Cellular Immunology*.

[61] Ribeiro A., Laranjeira P., Mendes S. (2013). Mesenchymal Stem Cells From Umbilical Cord Matrix, Adipose Tissue and Bone Marrow Exhibit Different Capability to Suppress Peripheral Blood B, Natural Killer and T Cells. *Stem Cell Research & Therapy*.

[62] Deuse T., Stubbendorff M., Tang-Quan K. (2011). Immunogenicity and Immunomodulatory Properties of Umbilical Cord Lining Mesenchymal Stem Cells. *Cell Transplantation*.

[63] Amable P. R., Teixeira M. V. T., Carias R. B. V., Granjeiro J. M., Borojevic R. (2014). Protein Synthesis and Secretion in Human Mesenchymal Cells Derived From Bone Marrow, Adipose Tissue and Wharton’s Jelly. *Stem Cell Research & Therapy*.

[64] Sabapathy V., Sundaram B., Sreelakshmi V. M., Mankuzhy P., Kumar S. (2014). Human Wharton’s Jelly Mesenchymal Stem Cells Plasticity Augments Scar-Free Skin Wound Healing With Hair Growth. *PLoS ONE*.

[65] Kim D. W., Gazourian L., Quadri S. A., Sherr D. H., Sonenshein G. E. (2000). The RelA NF-*κ*B Subunit and the Aryl Hydrocarbon Receptor (AhR) Cooperate to Transactivate the c-Myc Promoter in Mammary Cells. *Oncogene*.

[66] Sekine H., Mimura J., Oshima M. (2009). Hypersensitivity of Aryl Hydrocarbon Receptor-Deficient Mice to Lipopolysaccharide-Induced Septic Shock. *Molecular and Cellular Biology*.

[67] Thurmond T. S., Staples J. E., Silverstone A. E., Gasiewicz T. A. (2000). The Aryl Hydrocarbon Receptor has a Role in the In Vivo Maturation of Murine Bone Marrow B Lymphocytes and Their Response to 2,3,7,8-Tetrachlorodibenzo-p-dioxin. *Toxicology and Applied Pharmacology*.

[68] Vaidyanathan B., Chaudhry A., Yewdell W. T. (2017). The Aryl Hydrocarbon Receptor Controls Cell-Fate Decisions in B Cells. *Journal of Experimental Medicine*.

[69] Al-Ghezi Z. Z., Singh N., Mehrpouya-Bahrami P., Busbee P. B., Nagarkatti M., Nagarkatti P. S. (2019). AhR Activation by TCDD (2, 3, 7, 8-Tetrachlorodibenzo-p-Dioxin) Attenuates Pertussis Toxin-Induced Inflammatory Responses by Differential Regulation of Tregs and Th17 Cells Through Specific Targeting by microRNA. *Frontiers in Microbiology*.

[70] Li X.-m., Peng J., Gu W., Guo X.-j. (2016). TCDD-Induced Activation of Aryl Hydrocarbon Receptor Inhibits Th17 Polarization and Regulates Non-Eosinophilic Airway Inflammation in Asthma. *PLoS ONE*.

[71] Vogel C. F., Wu D., Goth S. R. (2013). Aryl Hydrocarbon Receptor Signaling Regulates NF-*κ*B RelB Activation During Dendritic-Cell Differentiation. *Immunology and Cell Biology*.

[72] Kimura A., Abe H., Tsuruta S. (2014). Aryl Hydrocarbon Receptor Protects Against Bacterial Infection by Promoting Macrophage Survival and Reactive Oxygen Species Production. *International Immunology*.

[73] Memari B., Bouttier M., Dimitrov V. (2015). Engagement of the Aryl Hydrocarbon Receptor in Mycobacterium Tuberculosis-Infected Macrophages has Pleiotropic Effects on Innate Immune Signaling. *The Journal of Immunology*.

[74] Mezrich J. D., Fechner J. H., Zhang X., Johnson B. P., Burlingham W. J., Bradfield C. A. (2010). An Interaction Between Kynurenine and the Aryl Hydrocarbon Receptor can Generate Regulatory T Cells. *The Journal of Immunology*.

[75] Wisniewski H.-G., Hua J., Poppers D. M., Naime D., Vilcek J., Cronstein B. N. (1996). TNF/IL-1-Inducible Protein TSG-6 Potentiates Plasmin Inhibition by Inter-Alpha-Inhibitor and Exerts a Strong Anti-Inflammatory Effect in Vivo. *Journal of Immunology*.

[76] Chirumbolo S. (2010). The Role of Quercetin, Flavonols and Flavones in Modulating Inflammatory Cell Function. *Inflammation & Allergy - Drug Targets*.

[77] Mohammadi-Bardbori A., Bengtsson J., Rannug U., Rannug A., Wincent E. (2012). Quercetin, Resveratrol, and Curcumin are Indirect Activators of the Aryl Hydrocarbon Receptor (AHR). *Chemical Research in Toxicology*.

[78] Ciolino H. P., Daschner P. J., Yeh G. C. (1999). Dietary Flavonols Quercetin and Kaempferol are Ligands of the Aryl Hydrocarbon Receptor That Affect CYP1A1 Transcription Differentially. *Biochemical Journal*.

[79] Wang X., Xie X., Li Y. (2024). Quercetin Ameliorates Ulcerative Colitis by Activating Aryl Hydrocarbon Receptor to Improve Intestinal Barrier Integrity. *Phytotherapy Research*.

[80] D’Esposito V., Ambrosio M. R., Liguoro D. (2021). In Severe Obesity, Subcutaneous Adipose Tissue Cell-Derived Cytokines are Early Markers of Impaired Glucose Tolerance and are Modulated by Quercetin. *International Journal of Obesity*.

[81] Almajwal A. M., Alam I., Abulmeaty M., Razak S., Pawelec G., Alam W. (2020). Intake of Dietary Advanced Glycation End Products Influences Inflammatory Markers, Immune Phenotypes, and Antiradical Capacity of Healthy Elderly in a Little-Studied Population. *Food Science & Nutrition*.

[82] Yao D., Wang S., Wang M., Lu W. (2018). Renoprotection of Dapagliflozin in Human Renal Proximal Tubular Cells via the Inhibition of the High Mobility Group Box 1-Receptor for Advanced Glycation End Products-Nuclear Factor-*κ*B Signaling Pathway. *Molecular Medicine Reports*.

[83] Zhao Y., Zhang L., Qiao Y. (2013). Heme Oxygenase-1 Prevents Cardiac Dysfunction in Streptozotocin-Diabetic Mice by Reducing Inflammation, Oxidative Stress, Apoptosis and Enhancing Autophagy. *PLoS ONE*.

[84] Yin M., Zhang Y., Yu H., Li X. (2021). Role of Hyperglycemia in the Senescence of Mesenchymal Stem Cells. *Frontiers in Cell and Developmental Biology*.

[85] Saki N., Jalalifar M. A., Soleimani M., Hajizamani S., Rahim F. (2013). Adverse Effect of High Glucose Concentration on Stem Cell Therapy. *International Journal of Hematology-Oncology and Stem Cell Research*.

[86] Lee H. J., Chae C. W., Han H. J. (2023). Enhancing the Therapeutic Efficacy of Mesenchymal Stem Cell Transplantation in Diabetes: Amelioration of Mitochondrial Dysfunction-Induced Senescence. *Biomedicine & Pharmacotherapy*.

[87] Mbongue J. C., Nicholas D. A., Torrez T. W., Kim N.-S., Firek A. F., Langridge W. H. (2015). The Role of Indoleamine 2, 3-Dioxygenase in Immune Suppression and Autoimmunity. *Vaccines*.

[88] Larsen M. C., Rondelli C. M., Almeldin A. (2023). AhR and CYP1B1 Control Oxygen Effects on Bone Marrow Progenitor Cells: The Enrichment of Multiple Olfactory Receptors as Potential Microbiome Sensors. *International Journal of Molecular Sciences*.

[89] Shimada T., Sugie A., Shindo M. (2003). Tissue-Specific Induction of Cytochromes P450 1A1 and 1B1 by Polycyclic Aromatic Hydrocarbons and Polychlorinated Biphenyls in Engineered C57BL/6J Mice of Arylhydrocarbon Receptor Gene. *Toxicology and Applied Pharmacology*.

[90] Carrera A. N., Grant M. K. O., Zordoky B. N. (2020). CYP1B1 as a Therapeutic Target in Cardio-Oncology. *Clinical Science*.

[91] Zordoky B. N., El-Kadi A. O. (2009). Role of NF-*κ*B in the Regulation of Cytochrome P450 Enzymes. *Current Drug Metabolism*.

[92] Theken K. N., Deng Y., Kannon M. A., Miller T. M., Poloyac S. M., Lee C. R. (2011). Activation of the Acute Inflammatory Response Alters Cytochrome P450 Expression and Eicosanoid Metabolism. *Drug Metabolism and Disposition*.

[93] Choi E. J., Kim T., Kim G.-H. (2012). Quercetin Acts as an Antioxidant and Downregulates CYP1A1 and CYP1B1 Against DMBA-Induced Oxidative Stress in Mice. *Oncology Reports*.

[94] Vrba J., Kren V., Vacek J., Papouskova B., Ulrichova J. (2012). Quercetin, Quercetin Glycosides and Taxifolin Differ in Their Ability to Induce AhR Activation and CYP1A1 Expression in HepG2 Cells. *Phytotherapy Research*.

[95] Friberg M., Behndig A. F., Bosson J. A. (2023). Human Exposure to Diesel Exhaust Induces CYP1A1 Expression and AhR Activation Without a Coordinated Antioxidant Response. *Particle and Fibre Toxicology*.

[96] Zhang Z., Liew C. W., Handy D. E. (2010). High Glucose Inhibits Glucose-6-Phosphate Dehydrogenase, Leading to Increased Oxidative Stress and *β*-Cell Apoptosis. *The FASEB Journal*.

